# Risk predict model using multi-drug resistant organism infection from Neuro-ICU patients: a retrospective cohort study

**DOI:** 10.1038/s41598-023-42522-2

**Published:** 2023-09-15

**Authors:** Hu Jiang, Hengping Pu, Nanqu Huang

**Affiliations:** 1 Nursing Department, The Third Affiliated Hospital of Zunyi Medical University (The First People’s Hospital of Zunyi), Zunyi, 563000 Guizhou China; 2 Drug Clinical Trial Institution, The Third Affiliated Hospital of Zunyi Medical University (The First People’s Hospital of Zunyi), Zunyi, 563000 Guizhou China

**Keywords:** Microbiology, Diseases, Medical research, Risk factors

## Abstract

The aim of this study was to analyze the current situation and risk factors of multi-drug-resistant organism (MDRO) infection in Neuro-intensive care unit (ICU) patients, and to develop the risk predict model. The data was collected from the patients discharged from Neuro-ICU of grade-A tertiary hospital at Guizhou province from January 2018 to April 2020. Binary Logistics regression was used to analyze the data. The model was examined by receiver operating characteristic curve (ROC). The grouped data was used to verify the sensitivity and specificity of the model. A total of 297 patients were included, 131 patients infected with MDRO. The infection rate was 44.11%. The results of binary Logistics regression showed that tracheal intubation, artery blood pressure monitoring, fever, antibiotics, pneumonia were independent risk factors for MDRO infection in Neuro-ICU (*P* < 0.05), AUC = 0.887. The sensitivity and specificity of ROC curve was 86.3% and 76.9%. The risk prediction model had a good predictive effect on the risk of MDRO infection in Neuro ICU, which can evaluate the risk and provide reference for preventive treatment and nursing intervention.

## Introduction

Intensive care unit (ICU) is a gathering place for patients with acute and critical illness. In recent years, with the increase in the incidence of stroke and traumatic brain injury (TBI), Neuro-ICU has gradually developed and become an ICU specialized in treating neurological problems^1,2^. Due to the higher antibiotic use rate and more invasive treatments in critically ill patients, the problem of multi-drug-resistant organism (MDRO) has become more prominent^3–5^.

Multidrug-resistant organisms refer to organisms resistant to one or more classes of antimicrobials, and these bacteria are often resistant to most available antimicrobial agents^6,7^. MDRO infection is complex and difficult to treat, and it is easy to cause an outbreak of nosocomial infection. MDRO nosocomial infection not only prolongs the number of hospitalization days for patients, but also increases the hospitalization costs of patients. These are bringing a huge economic burden to patients, and more serious nosocomial infection in the ICU affects the success rate of critically ill patients and increases the mortality rate of ICU patients^8,9^. Therefore, the management of MDRO has gradually become an important part of hospital infection control.

Due to its particularity, Neuro-ICU patients have longer recovery time and longer hospital stay. Studies have shown that the hospital stay of TBI patients was about 19.4 ± 13.9 days^10^. Studies have shown that there is a correlation between the length of stay in the ICU and the infection rate of MDRO^11^, the prevalence of infections acquired in Intensive Care Units (ICU) is higher than it is in other hospital units^12^, ICU MDRO infection rate can be as high as 50%^13^. The incidence of MDRO infections in the neuro ICU is high^14^, but studies are still relatively limited. Therefore, the prevention and control of Neuro-ICU MDRO infection become an urgent problem to be solved, and prevention is the most economical and effective way to control MDRO infection.

The purpose of this study was to explore the high-risk factors for MDRO infection, establish a Neuro-ICU MDRO nosocomial infection transmission risk model, propose targeted infection prevention and control measures, and reduce the transmission and infection of MDRO in a timely and effective manner, thus preventing an outbreak of MDRO infection.

## Methods

### Study design and population

Risk factors related to infection of multi-drug-resistant organism in Neuro-ICU patients were investigated by searching Chinese and foreign literatures, consulting critical care experts, conducting a group discussion, designing and modifying the data collection items, including: ①General demographic information, such as gender, age, etc. ②Treatment Circumstances, such as invasive operation, level of consciousness, etc. ③Laboratory indicator and the patient's own physical conditions. Researchers grouped patients by whether they were infected or colonized with multi-drug resistant organism.

**Figure 1:**
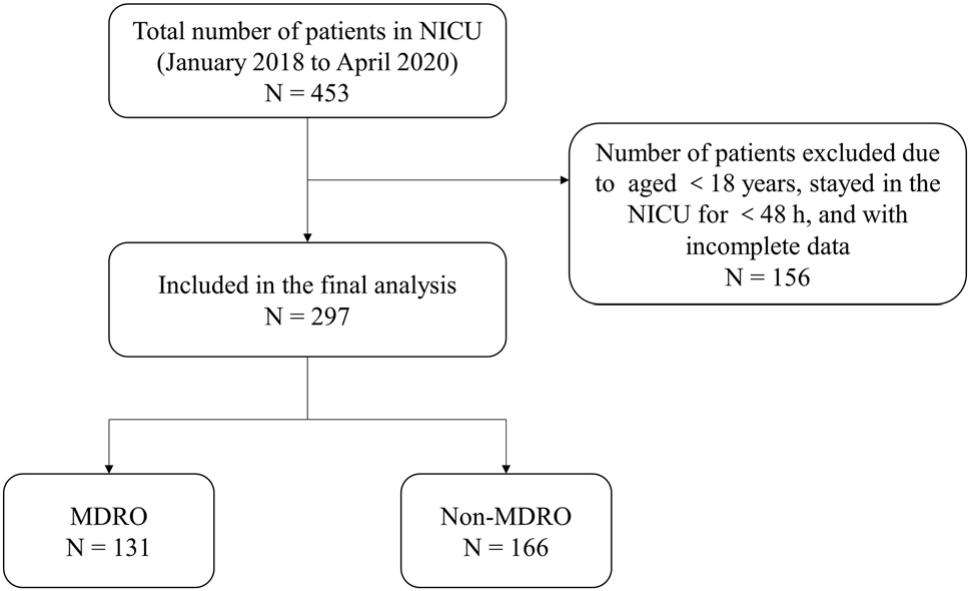
Patient enrollment flow chart.

The study was a single-center, retrospective study. The cluster sampling method was used to collect relevant data of all consecutive patients of Neuro-ICU in the Third Affiliated Hospital of Zunyi Medical University from January 1, 2018 to April 30, 2020. Inclusion criteria included: ①Patients aged ≥ 18 years. ②Patients staying in the ICU for ≥ 48 h. ③Patients who were transferred to the general ward after being transferred from the ICU. Exclusion criteria: patients with incomplete data or an MDRO infection has occurred. In this study, univariate and multivariate analysis were used to screen risk factors. A total of 297 patients were included in this study (Fig. 1).

### Data Collection

In this study, we retrospectively investigated the hospital's Neuro-ICU patient entry and exit registration form, electronic medical record system, nursing document system, and medical record file to obtain the required information. All data were collected before the diagnosis of MDRO infection, GCS score and albumin level were collected as the first test results of patients transferred to ICU. Combination of antibiotics refers to patients received two or more antibiotics at the same time. Antibiotics are drugs used to treat various bacterial or infections inhibit infection by pathogenic microorganisms, including all antibiotics such as sulfonamides, quinolones and β-lactam antibiotics. All data were double-entered using Excel2016 software. To determine whether patients are infected with MDROs, aseptically collected sputum, blood, cerebrospinal fluid, and midstream urine samples were collected from them conventionally. The criteria for determining MDROs have been developed according to the 2010 international standardized definition of MDROs jointly proposed by the United States and other countries^15^.

### Ethical considerations

This study was approved by the Ethics Committee of The Third Affiliated Hospital of Zunyi Medical University (The First People’s Hospital of Zunyi) (2017–215). As the patient information was anonymized and deidentified before analysis, the ethics committee of the First People’s Hospital of Zunyi waived the need for informed consent. And all methods were performed following the Declaration of Helsinki.

### Statistical analysis

SPSS 20.0 software was used for statistical processing, measurement data was expressed by independent sample* t* test or non-parametric rank sum test, count data was expressed by frequency and rate, and chi-square test was used; binary Logistics regression was used to obtain risk factors for MDRO infection and build a prediction model, the ROC curve was drawn with sensitivity as the ordinate and (1-specificity) as the abscess coordinate, and the prediction effect of the model was verified. *P* < 0.05 indicated that the difference was statistically significant.

### Ethical approval and consent to participate

This research was approved by the Ethics Committee of the The Third Affiliated Hospital of Zunyi Medical University (The First People’s Hospital of Zunyi) (2017-215). As the patient information was anonymized and deidentified before analysis, informed consent was not needed.

## Results

### General data and MDRO infection

This study was included 297 patients’ information, including 206 patients (69.4%), 91 female (30.7%), age distribution at 18–95 (56.79 ± 16.057), of which 131 patients have MDRO infections. The infection rate was 44.11%. Among the 131 patients infected, 106 (80.92%) were infected with 1 bacterium, 25 (19.08%) infected 2 and more bacteria. The top five bacteria were detected as *Acinetobacter baumannii* (44, 33.59%), *Klebsiella pneumoniae* (39, 29.77%), *Pseudomonas pyocyaneum* (22, 16.79%), Methicillin-resistant *Staphylococcus aureus* (8, 6.1%), *Escherichia coli* (7, 5.34%).

### Construction of risk prediction model

In this study, 297 patients were used for the construction of risk prediction models. The results of single factor analysis showed that the difference between age, medical insurance, admission, diagnosis between two groups were significant, and the remaining factors were statistically significant (*P* < 0.05) (Table 1). In contrast, the variable was used as a variable in a single factor analysis, which uses binary logistics regression analysis. The results show that tracheal intubation, artery blood pressure monitoring, fever, antibiotics, pneumonia were found as independent risk factors of multiple drug-resistant bacteria infections, predictive model formulas: P = 1/1 + exp (−Z). This prediction model was used as z = 1.815 × tracheal inhibition + 2.012 × artery blood pressure monitoring + 1.650 × fever + 2.087 × antibiotics + 1.750 × pneumonia + (-0.83 × albumin) (Table 2).

**Table 1:** Differences between groups (n = 297).

Variable	MDRO (n = 131)	Non-MDRO (n = 166)	*t*/χ^2^	*P*
Age (year)	57.20 ± 13.52	56.48 ± 17.84	− 0.397	0.692
Male	99 (75.57)	107 (64.46)	4.236	0.039
Diagnosis			3.339	0.503
Traumatic brain injury	40 (30.54)	51 (30.72)		
Stroke	75 (57.25)	86 (51.81)		
Brain tumor	6 (4.58)	8 (4.82)		
Epilepsy	4 (3.05)	13 (7.83)		
Other	6 (4.58)	8 (4.82)		
GCS score			13.301	0.001
13–15	72 (55.0)	45 (27.1)		
9–12	42 (32.1)	61 (36.7)		
< 8	17 (13)	60 (36.1)		
Tracheal intubation	119 (90.84)	74 (44.58)	69.645	＜0.001
Tracheotomy	55 (41.98)	19 (11.45)	36.501	＜0.001
Artery blood pressure monitoring	126 (96.18)	138 (83.13)	12.627	＜0.001
Deep venous	75 (57.25)	63 (37.95)	10.964	0.001
Mechanical ventilation	93 (70.99)	57 (34.34)	39.354	＜0.001
Sedation	89(67.94)	80(48.19)	11.641	0.001
Analgesia	65 (49.62)	50 (30.12)	11.731	0.001
Fever	124 (94.66)	81 (48.80)	72.026	＜0.001
Blood transfusion	72 (54.96)	52 (31.33)	16.820	＜0.001
Surgery	82 (62.60)	78 (46.99)	7.177	0.007
Antibiotics	127 (96.95)	108 (65.06)	45.070	＜0.001
Combination of antibiotics	55 (41.98)	28 (16.87)	22.940	＜0.001
Pneumonia	117 (89.31)	93 (56.02)	39.174	＜0.001
Albumin (g/L)	28.83 ± 4.83	32.33 ± 4.96	6.107	＜0.001

**Table 2:** Multiple logistic regression of MDRO infection (n = 297).

Risk factors	B	SE	Wald	*P*	OR (95% CI)
Tracheal intubation	1.815	0.419	18.767	＜0.001	6.140 (2.701–13.955)
Artery blood pressure monitoring	2.012	0.599	11.673	0.001	7.479 (2.358–23.723)
Fever	1.650	0.491	11.313	0.001	5.209 (1.991–13.626)
Antibiotics	2.087	0.635	10.811	0.001	8.064 (2.324–27.984)
Pneumonia	1.750	0.402	18.956	＜0.001	5.755 (2.617–12.652)
Albumin	-0.83	0.033	6.373	0.012	0.920 (0.862–0.982)
Constant	-5.320	1.479	12.944	＜0.001	

### Risk prediction model fitting test

**Figure 2:**
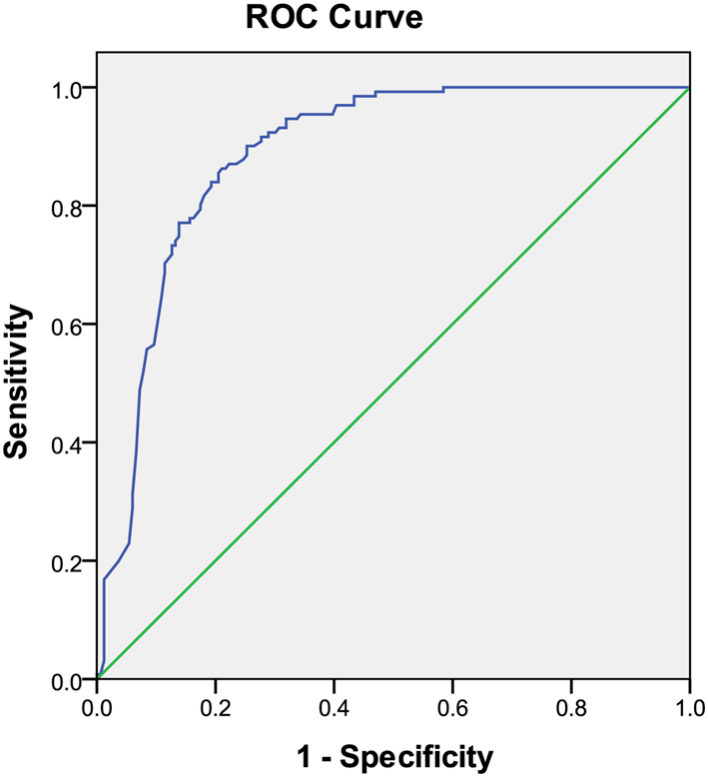
ROC curve of MDRO infection prediction model.

The results of HOSMER and LEMESHOW test (*P* = 0.831) were indicated that the model can better interact with the actual occurrence of MDRO infections. The truncation value was calculated by the predictive model formula used as the test variable, and whether it returns to the state variable ROC curve. Figure 2 showed the area of the ROC curve (AUC) = 0.887, 95% CI (0.849, 0.925), *P* < 0.001 was indicated the predicted effect of this prediction model was very good. In this study, the maximum value of Youden index was used to judge the optimal critical value of the predictive model, the maximum value of Youden index was about 0.652, and the sensitivity was of ROC curve was 86.3%, the specificity was 78.9%, and the cutoff value was 0.476.

## Discussion

This study aimed to clarify the influencing factors of MDRO infection in Neuro-ICU, and further construct predict models according to the influencing factors. A total of 297 patients admitted in Neuro-ICU were included in this study, our analysis identified risk factors for MDRO infection, and we developed predictive models using these risk factors.

Our study found that *Acinetobacter baumannii, Klebsiella pneumoniae,* and *Pesudomonas pyocyaneum* were the most common pathogens in Neuro-ICU. Recent study showed that *Acinetobacter baumannii* was found as most frequent bacteria^16,17^. Among nosocomial infection and colonization, 76% of the bacterial infections were due to Gram-negative bacteria. *A. baumannii*, Enterobacteriaceae species and *P. aeruginosa* strains were the main isolated bacteria^12^. Yang et al.^18^ also reported that gram negative (GN) bacteria were more common than gram positive (GP) bacteria predominated in central nervous system (CNS) infections, and our study was a little different from previous research results. *Pseudomonas pyocyaneum* was found as one of the main bacteria in Neuro-ICU. Our results showed that the incidence of MRSA was low, this was similar to the research results of El Mekes et al.^12^. There are many studies related to ICU MDRO infection rate, and the results have large value fluctuation range, a Chinese study stated MDRO prevalence rate on admission in ICU was 30.5%^19^.

The present study found that MDRO infections were not uncommon in Neuro-ICU, although this study did not clarify the specific MDRO infection rate, among the study subjects included, the number of patients infected or colonized with MDRO has exceeded 44%, which is extremely high. However, this data did not represent the overall situation of Neuro-ICU. The subjects of our study were patients who entered the ICU and were transferred out. Some patients had been transferred to other hospitals or died during the ICU hospitalization process, so in this study we could not calculate the exact infection rate. However, there is still insufficient evidence for the MDRO infection rate in the Neuro ICU, and more studies are needed to confirm it.

Our study resulted in good predictive models with accuracies of 86.3% using logistic regression. In our study, we found that tracheal intubation, artery blood pressure monitoring, fever, antibiotics, pneumonia are significant predictors for MDRO infection. The use of antibiotics (OR: 8.064) was the most important factor in MDRO infection. In the current state of knowledge, there are few predictive models of MDROs infections in neuro-intensive care units. Minhas et al.^20^ modeled prediction through decision trees, and our predictive efficacy is similar to this study. Our study differed significantly from another Chinese study^21^, which examined a model to predict pulmonary infection of multidrug-resistant *Acinetobacter baumannii* in Neuro-ICU. This may be because we pooled and analyzed a variety of infections rather than pulmonary infection in our study.

The amount of antibiotic use of severe patients is significantly higher than those of ordinary ward. Due to the resistance or infection, antibiotics are often used in combination. It was found that antibiotics and combination of antibiotics are related to MDRO infection in univariate analysis. However, only antibiotic therapy is an independent risk factor of MDRO infection in the multiple logistic regression model. In Neuro-ICU, the majority patient's primary disease types are traumatic brain injury or stroke, and the pneumonia incidence of two diseases is high^22,23^. Using mechanical ventilation will aggravate the incidence of pneumonia^24^.

The use of antibiotics to treat pneumonia is prone to the occurrence and propagation of multi-drug resistant organism^25–27^. A Meta-analysis has clearly been applying antibiotics in advance to risk factors for MDRO infection^28^. Our research further verified this view. In order to increase the cure rate of multi-drug bacteria, medical staff tend to use antibiotics to enhance the utility of drugs^27^. It may exacerbate this process. In addition, in this study, fever was also one of the risk factors of MDRO infection, critically ill patients may have a variety of reasons, and fever may be caused by patient's pneumonia. Among the patients with MDRO infection or planting, 89.31% of patients had pneumonia, while non-MDRO patients were also as high as 56.02%.

Recent studies have shown that the severity of trauma does not affect the incidence of MDRO, the incidence of MDRO in severely injured patients is lower than in ICU^29–31^. The type of admission and disease can indicate the severity of patients, in our results, GCS scores cannot ultimately predict MDRO infection, so our research indicates that MDRO infection is not associated with the severity of disease, this may be caused by autoimmune and early application of antibiotics in patients with acute trauma^32^.

The results for risk factors were consistent with previous studies in the use of invasive operation^24,33,34^. In our study, persistent artery blood pressure monitoring and tracheal intubation were two invasive appliances associated with MDRO infection, such invasive operations destroy normal physiological integrity and local immune function. Our research results were different from the past. In the past, urinary tube and feeding tubes were the main indwelling instruments of MDRO infection^34,35^. Due to the particularity of neurological patients, almost all patients will remain in these two types of piping, so we did not incorporate these two invasive operations.

Furthermore, we found an association between positive MDRO and albumin level, there is a literature pointed out that the lower the serum albumin level, the higher the risk of patient infection^36^, our research further verified this conclusion. The average age of the patients included in our study was older, and both the patients themselves and the disease caused malnutrition and hypoproteinemia. Despite the study showed allogenic blood transfusions cause immune modulation and have a negative influence on the immune system and may result in increased infections with MDRO^37^. However, blood transfusion still cannot be used as a predictive variable, although patients with anemia or low nutritious states are worthy of attention, because this type of patient is a high-risk population of MDRO infection.

However, in our study, there were no correlation between the gender, age, medical insurance with MDRO infection. There were many studies had shown that age and gender were not associated with acquired MDRO^38,39^. A recent research indicated MDRO that infections were numerically associated with the female sex, greater age, and comorbidities^40^. These factors may require more research to further confirm the correlation.

Future clinical practice should reinforce multidisciplinary coordination so that physicians, pharmacists, and nurses play a more active role in patient care. The screening of patients for multidrug-resistant bacteria in the neurologic intensive care unit should be a routine part of the treatment of patients after admission. In the process of stepping and standardizing the medication regimen of patients, pharmacists should serve as a guide. Physicians and pharmacists will work together in the future to pursue and develop therapeutic medications^41–43^. Nurses are essential components of clinical practice and should play a full role in preventing MDROs and managing them on a daily basis. A high incidence of MDROs in Neuro-ICU makes multidisciplinary work necessary for effective prevention and management of MDRO infections.

There were several limitations in our study. First, this was a retrospective study of a single center, the researchers only collected patient data in a tertiary, a hospital in one province, and the data cannot represent the actual situation in the region or the whole country, and there may be bias. Second, there were less research samples, compared with the comprehensive ICU, Neuro-ICU has a smaller patient population, and the researchers only collected data for the past two years. Finally, although there was established a predictive model based on risk factors, there was no further verification of prediction model, and the impact of the risk prediction model on the actual incidence of MDRO infection in Neuro-ICU patients was not investigated.

## Conclusion

**Figure 3:**
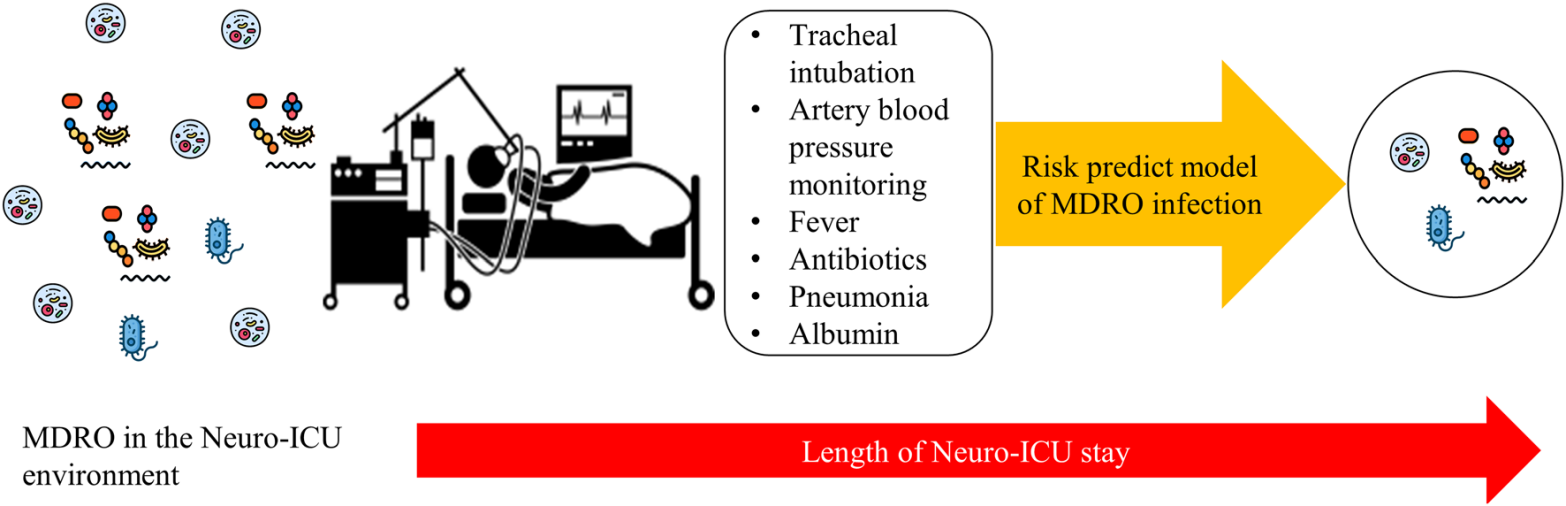
A simplified schematic diagram representing risk predict model using multi-drug resistant organism infection from Neuro-ICU patients.

This study constructed a predictive model with 6 variables, and the patients with higher risk would be screened (Fig. 3). The establishment of an ICU MDRO infection risk prediction model can eliminate the blindness of MDRO infection or colonization treatment, so that medical staff can more rationally and effectively use relevant resources, and timely, accurately and comprehensively grasp the changes of patients. Targeted implementation of preventive measures for patient use, and ultimately prevent MDRO nosocomial infection.

### Supplementary Information

Supplementary Information.

## Data Availability

The original contributions presented in the study are included in the supplementary material, further inquiries can be directed to the corresponding author.
